# NIC_6_-TT Vaccine Reduces Nicotine-Seeking Behavior and Expression of Nicotine-Induced Locomotor Sensitization in Rats

**DOI:** 10.3390/brainsci15040364

**Published:** 2025-03-31

**Authors:** Susana Barbosa Méndez, Alberto Salazar-Juárez

**Affiliations:** Subdirección de Investigaciones Clínicas, Laboratorio de Neurofarmacología Conductual, Microcirugía y Terapéutica Experimental, Instituto Nacional de Psiquiatría, México DF 14370, Mexico; schefiel12@yahoo.com

**Keywords:** active vaccination, nicotine, antibodies, NIC_6_-TT vaccine, nicotine self-administration, nicotine locomotor sensitization

## Abstract

**Introduction**: Various models of nicotine vaccines have been evaluated. In humans, antibody levels are low and variable. In this sense, it is necessary to improve or optimize the nicotine vaccines already evaluated. We reported the efficacy of the M_6_-TT vaccine. Recently, we reported the efficacy of the COC-TT vaccine, which was developed from the M_6_-TT vaccine. Both vaccines generate high titers of antibodies and attenuate heroin- or cocaine-induced behavioral effects in rodents. **Aims and Methods**: The objective of this study was to determine whether the antibodies generated by a tetanus toxoid-conjugated nicotine vaccine (NIC_6_-TT) can produce anti-nicotine antibodies and decrease the nicotine-induced reinforcing and psychomotor effects. Male Wistar rats were immunized with the NIC_6_-TT. A solid-phase antibody-capture ELISA was used to monitor antibody titer responses after each booster dose in vaccinated animals. The study used nicotine self-administration and nicotine locomotor sensitization testing to evaluate the nicotine-reinforcing and psychomotor effects. **Results**: The NIC_6_-TT vaccine could generate high and sustained levels of anti-nicotine antibodies. The antibodies reduced the nicotine self-administration and expression of nicotine locomotor sensitization. **Conclusions**: These findings suggest that the NIC_6_-TT vaccine generates a robust immunogenic response capable of reducing the reinforcing and psychomotor effects of nicotine, which supports its possible future use in clinical trials for the treatment of smokers. **Implications**: Smoking is the second most used psychoactive substance in the world, which is associated with millions of preventable deaths. An effective treatment is required. Nicotine vaccines must generate high levels of anti-nicotine antibodies, but above all, the decay curve of the antibodies must be very slow, so that they can provide long-term protection and support long-term smoking abstinence. The NIC_6_-TT vaccine meets these properties.

## 1. Introduction

Tobacco smoking is currently considered a serious global public health problem [1]. Clinical and epidemiological evidence indicates that (1) smoking is the second most used psychoactive substance in the world (with more than 1.5 billion smokers worldwide, that is, almost 23% of the world’s population are tobacco users); and (2) smoking is considered a preventable risk factor associated with the development of respiratory, cardiovascular, and metabolic diseases, as well as several types of human cancers [2].

Pioneering neurobiological studies have demonstrated that nicotine is the main psychoactive component of tobacco, and nicotine is considered to play a key role in tobacco dependence, as the activation of nicotinic acetylcholine receptors (nAChR) by nicotine generates the tobacco-induced reinforcing effects [3].

Given the key role of nicotine in smoking and the health problems associated with tobacco abuse, it is essential to develop therapeutic strategies aimed at reducing the nicotine-induced reinforcing effects, indirectly reducing tobacco dependence, and reducing the harm associated with smoking.

In this sense, various studies demonstrated the effectiveness of the 6-(carboxymethylureido)-(+/−)-nicotine linked to keyhole limpet hemocyanin (CMUNic-KLH) or trans-3-aminomethylnicotine linked to recombinant Pseudomonas aeruginosa exoprotein A (NIC-rEPA) vaccines in generating specific anti-nicotine antibodies, capable of capturing nicotine molecules in the blood and preventing nicotine from crossing the blood–brain barrier [4,5,6], thus reducing the toxic effects of nicotine [7], the nicotine-induced increase in dopamine release in the nucleus accumbens shell (NAC Shell) [8], nicotine self-administration [9,10,11], and nicotine-induced locomotor activity [12,13], in rodents and primates.

These studies support the potential feasibility that active vaccination could be a good therapeutic tool for the treatment of the nicotine-reinforcing effects in the context of smoking cessation programs.

In humans, several vaccines have been evaluated—Nic-Qb (NIC002), NicVax (3′AmNic-rEPA), Niccine (nicotine hapten tetanus-toxoid), and TA-NIC—in clinical trials, phases II and III [14,15,16,17]. Studies agree that these vaccines are safe and well tolerated by patients [18,19,20] and that they can generate antibodies capable of recognizing nicotine. However, their usefulness has been limited due to the side effects they produce (flu-like symptoms, pain at the injection site, headache, among others) but mainly to the amount of antibodies that this therapy produces [21,22,23]. These studies report that the levels of antibodies produced by these vaccines are low (inadequate to reduce the nicotine-reinforcing effect) and variable, showing an important dependence on the individual [24,25,26,27,28].

To address these limitations, many laboratories have directed efforts to evaluate the effect of various methodological strategies aimed at improving vaccine synthesis processes and increasing their efficiency. Among these strategies are the improvement and optimization of the hapten and its exposure (localization, density, clustering, conformational restriction), the evaluation of different spacer arms, carrier proteins, adjuvants, and administration routes, in rodents [29,30,31]. However, some of these vaccines are not suitable for use in humans, mainly because the structural elements that are used for their synthesis have not been approved for human use [32,33]. Additionally, although these improvements in some cases increased antibody levels, the results remain inconsistent [29,30,31,32,33]. Therefore, it is necessary to find a vaccine that is safe and effective in reducing the reinforcing effects of nicotine.

We previously demonstrated the effectiveness of the morphine vaccine conjugated with tetanus toxoid (M_6_-TT) and formulated with aluminum hydroxide as an adjuvant [34,35,36,37,38,39,40,41]. This vaccine was able to generate high titers of antibodies capable of capturing morphine, heroin, and their metabolites [34,35] and reducing antinociception [36,37,38,39], self-administration [34,35], and locomotor activity [39,40] induced by heroin or morphine, in Wistar rats and *BALB/c*, *C57Bl/6*, and *DBA/2* mice [37]. Recently, our laboratory reported the efficacy of a vaccine against cocaine (COC-TT), structurally related to the M_6_-TT vaccine [41]. In this study, we maintained the length and chemical nature of the spacer arm, the carrier protein, and the adjuvant as essential structural elements to preserve the immunogenic and immunoprotective properties of the M_6_-TT vaccine; only the hapten was changed (cocaine to morphine). The results showed that the COC-TT vaccine, like the M_6_-TT vaccine, generated high levels of cocaine-specific antibodies. These antibodies reduced cocaine self-administration and place preference, as well as cocaine-induced *Fos* protein expression [41].

Thus, (1) given that it is necessary to optimize vaccines against nicotine, (2) given that various studies suggest that the magnitude of the immunogenic response and the specificity of the antibodies depends on the hapten, the carrier protein, the binding position of the hapten to the carrier protein and even the adjuvant with which the final formulation of the vaccine is made, and (3) given that the M_6_-TT and COC-TT vaccines, structurally similar, are capable of generating high titers of specific antibodies capable of reduce the reinforcing effects of morphine, heroin, or cocaine; it would be plausible to modify the M_6_-TT vaccine again, exclusively changing the hapten (nicotine for morphine) and maintaining the rest of the structural elements of the vaccine (spacer arm, carrier protein, and adjuvant), to maintain the immunogenic properties of the M_6_-TT and COC-TT vaccines (high antibody titers). Therefore, the objective of this study was to determine whether antibodies generated by a tetanus–nicotine toxoid conjugate (NIC_6_-TT) can recognize and capture nicotine and reduce the reinforcing effects of nicotine.

## 2. Methods

### 2.1. Subjects

The study used male Wistar rats weighing 250–280 g at the onset of the experiments (50 days old). They were housed four per cage in standard plastic rodent cages (57 cm × 35 cm × 20 cm) in a colony room at 21 ± 2 °C and 40–50% humidity, under a 12 h light/dark cycle (lights on at 7:00 AM). The animals had free access to water during the experiments. Rats trained in food self-administration were restricted to 20 g per day of rat chow after daily operant sessions. The rats were acclimated to the experimental chambers for 2 h over 5 days, before the lever-press training sessions. All the experiments took place during the light phase of the light/dark cycle (9:00 a.m. to 5:00 p.m.). All the animal studies and standards followed the Principles of Laboratory Animal Care, as outlined by the National Institutes of Health in the USA, and were approved by the Animal Care and Bioethics Committee of the National Institute of Psychiatry in Mexico City (CEI/C/IC092019.2/2012).

### 2.2. Drugs

Nicotine (nicotine tartrate salt; Sigma-Aldrich, St. Louis, MO, USA) was purchased from the Sigma-Aldrich company. Cocaine hydrochloride was kindly donated by the Mexican government, and both drugs were under strict regulatory controls. All the drugs used in experimental animals were placed under official surveillance (COFEPRIS-LC-0004-2003).

Nicotine tartrate was dissolved in sodium phosphate buffer (pH 7.2–7.4). All nicotine solutions were adjusted to pH 7.4 using sodium hydroxide (1 M). Cocaine hydrochloride was dissolved in sterile saline solutions (0.9% NaCl, Sigma Aldrich). All solutions were prepared, kept aliquoted, filtered through a 25 µm syringe filter (Fisher Scientific, Pittsburgh, PA, USA), and frozen at −20 °C before use. Upon use, all reagents were thawed at room temperature and freshly prepared before dosing to animals. The nicotine solution was injected at a volume of 1 mL/kg body weight.

### 2.3. Synthesis of the NIC-TT Vaccine

The synthesis of the NIC-TT vaccine was performed using previously reported methods [4,5,33,40,42]. For details, see Supplementary Materials.

### 2.4. Determination of Serum Antibody Titers via ELISA

A standard protocol was used to perform the determination of serum antibody titers via ELISA (Supplementary Materials) [33,40].

### 2.5. Behavioral Procedure

#### 2.5.1. Self-Administration Procedure

The nicotine self-administration procedures were performed according to a method described elsewhere [43]. Briefly, the self-administration procedure was divided into three stages: (1) training or acquisition, (2) extinction, and (3) re-acquisition. At acquisition, the rats were trained to self-administer nicotine using a 0.04 mg/kg nicotine unit dose (30 μL/infusion/30 s), in daily 2 h sessions (FR5 with 2 min TO) for 6 days per week. In extinction, the experimental conditions were the same as during nicotine self-administration with the exception that pressing on the active lever resulted in the infusion of saline instead of nicotine. During the re-acquisition sessions, the nicotine or cocaine self-administration procedures were similar to those used during training for nicotine self-administration (FR5 with 2 min TO; complete methods in the Supplementary Materials).

#### 2.5.2. Behavioral Sensitization Procedure

A standard protocol was used to perform the sensitization procedure (Supplementary Materials) [44,45]. A standard protocol was used to perform the sensitization procedure [44,45]. Briefly, the animals were habituated to the activity chambers in three 30 min sessions and were randomly assigned to different pharmacological treatment groups. Locomotor activity was recorded for 30 min. The rats were returned to their home cages after each experimental session was completed (complete methods in the Supplementary Materials).

### 2.6. Immunization Schedule

A standard protocol was used to carry out animal immunization [33,40]. Briefly, in total, 50 rats were immunized with the NIC_6_-TT vaccine adsorbed to an aluminum hydroxide gel adjuvant (Pierce, Rockford, IL, USA), and 50 rats were immunized with the TT vaccine adsorbed to an aluminum hydroxide gel adjuvant. Each animal was injected subcutaneously (s.c.) at four sites over the shoulders bilaterally (two inoculations/side), for a total dose of NIC_6_-TT vaccine/adjuvant of 100 μg/1 mg aluminum hydroxide (≈100 μg/inoculation/animal/booster). Subsequently, 6 booster injections were administered using the same unit dose and adjuvant, over 14–16 weeks (once every 14 days). Control rats received TT injections at the same time as vaccinated rats. Fourteen days after each booster, the rats were bled, and their sera were collected and frozen at −20 °C until use.

### 2.7. Experimental Procedures

The study used 100 male Wistar rats, which were assigned to three experimental groups. For Experiment 1, we used 20 animals that were further divided into two experimental groups (n = 10); for Experiment 2, we used 40 animals that were further divided into two experimental groups (n = 20); for Experiment 3, we used 40 animals in two groups (n = 20). Each experimental group received a different pharmacological treatment.

#### 2.7.1. Experiment 1

Experiment 1 sought to characterize the anti-nicotine antibody titers produced by the NIC_6_-TT vaccine. This experiment was divided into two experimental phases: Phase I, the immunization phase, lasted 85 days, and Phase II, the titer maintenance phase, lasted 240 days.

**Figure 1:**
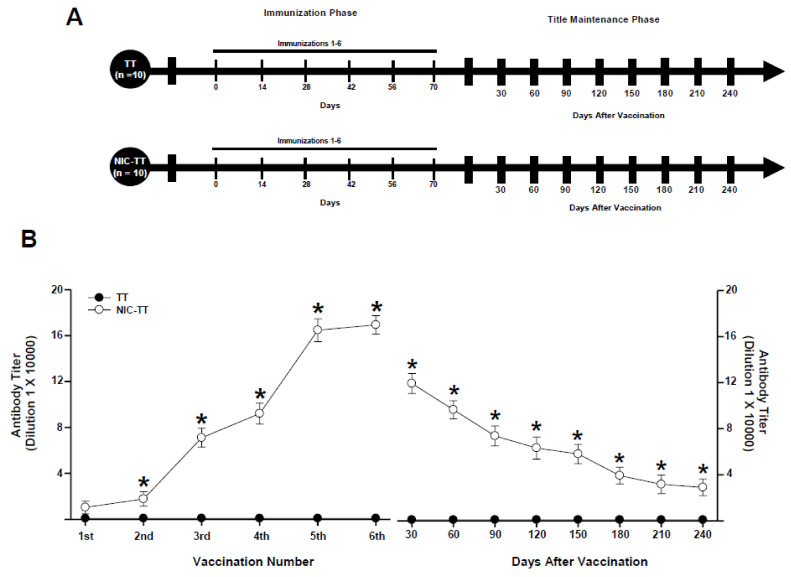
Experiment timeline (**A**). Antibody titer responses (to the sixth boost) in rats immunized with the TT or NIC_6_-TT vaccine (**B**). Time course of the kinetics of decay (**C**). Serum samples were collected 14 days after each immunization. Mean titers (±S.E.M.). * *p* < 0.01 indicates significant effects of the antibody titers generated by the NIC_6_-TT vaccine compared to the antibody titers generated by the TT vaccine in Wistar rats, as determined by two-way ANOVA followed by Tukey’s tests.

Two groups of animals were tested: TT (n = 10) and NIC_6_-TT (n = 10). Each group received a different pharmacological treatment (Figure 1A).

After three days of habituation to the laboratory, the rats (n = 20) were randomly assigned to the TT (n = 10) and NIC_6_-TT (n = 10) groups. During the immunization phase, the mice of the TT groups received immunization with the TT vaccine. In contrast, the animals in the NIC_6_-TT group received vaccination with the NIC_6_-TT vaccine, respectively. Fourteen days after each immunization, serum samples were taken to assess antibody titers. Both groups received 6 immunizations, one immunization every 14 days. In the titer maintenance phase, both groups were not immunized; however, serum samples were taken every 30 days to evaluate antibody titers.

#### 2.7.2. Experiment 2

This experiment focused on determining whether the NIC_6_-TT vaccine alters nicotine re-acquisition in animals that self-administered nicotine. This experiment was divided into three experimental phases: Phase I, the nicotine-acquisition phase, lasted 10 days. Phase II, the nicotine-extinction phase, lasted 85 days. This phase includes the immunization phase, which consists of 6 immunizations every 14 days. Phase III, the nicotine-re-acquisition phase, lasted 30 days. This phase includes the self-administration of different doses of nicotine (0.04 and 0.06 mg/kg) and cocaine (1 mg/kg).

Four groups of animals were tested: TT + SAL (n = 10); TT + NIC (n = 10); NIC_6_-TT + SAL (n = 10); and NIC_6_-TT + NIC (n = 10).

The TT + SAL and NIC_6_-TT + SAL groups received daily saline self-administration during the acquisition, extinction, and re-acquisition phases. During the extinction phase, rats in the TT group were immunized with the TT vaccine, and animals in the NIC_6_-TT group received the NIC_6_-TT vaccine.

**Figure 2:**
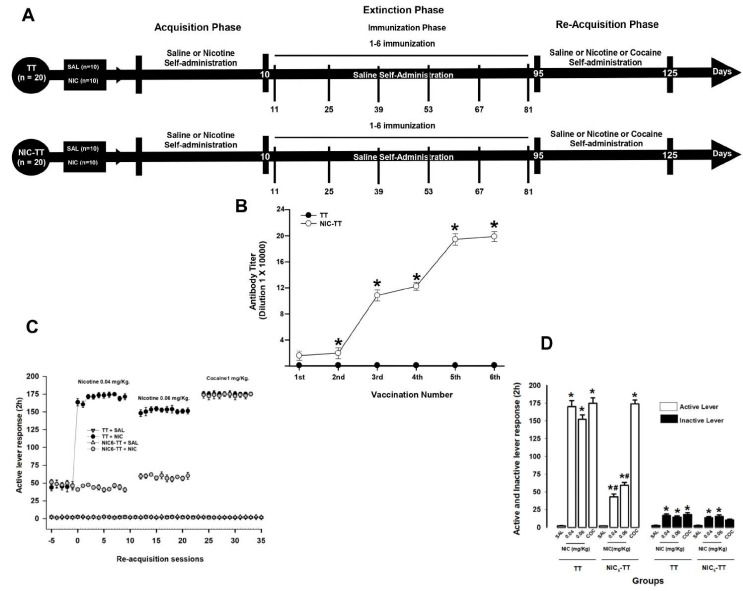
Experiment timeline (**A**). Antibody titer responses (to the sixth boost) in rats immunized with the TT or NIC_6_-TT vaccine (**B**). * *p* < 0.01 indicates significant effects of the antibody titers generated by the NIC_6_-TT vaccine following the sixth booster compared to the antibody titers generated by the TT vaccine in Wistar rats. The NIC_6_-TT vaccine reduces nicotine self-administration. Total nicotine seeking during re-acquisition (**C**). Mean responses on the active and inactive level during the re-acquisition period (**D**). * *p* < 0.01 indicates significant effects on the active and inactive lever responses in the TT + NIC and NIC_6_-TT + NIC groups compared to saline-treated groups. # *p* < 0.01 indicates significant effects on the active and inactive lever responses in the TT + NIC group compared to the NIC_6_-TT + NIC group, as determined by two-way ANOVA followed by Tukey’s tests.

In contrast, the TT + NIC and NIC_6_-TT + NIC groups received nicotine self-administration (0.04 mg/kg) during the acquisition. In the re-acquisition phase, the animals received self-administration of different doses of nicotine (0.04 and 0.06 mg/kg) and cocaine (1 mg/kg). In the extinction phase, both groups received daily saline self-infusion. During the extinction phase, rats in the TT group were immunized with the TT vaccine, and animals in the NIC_6_-TT group received the NIC_6_-TT vaccine (Figure 2A).

#### 2.7.3. Experiment 3

**Figure 3:**
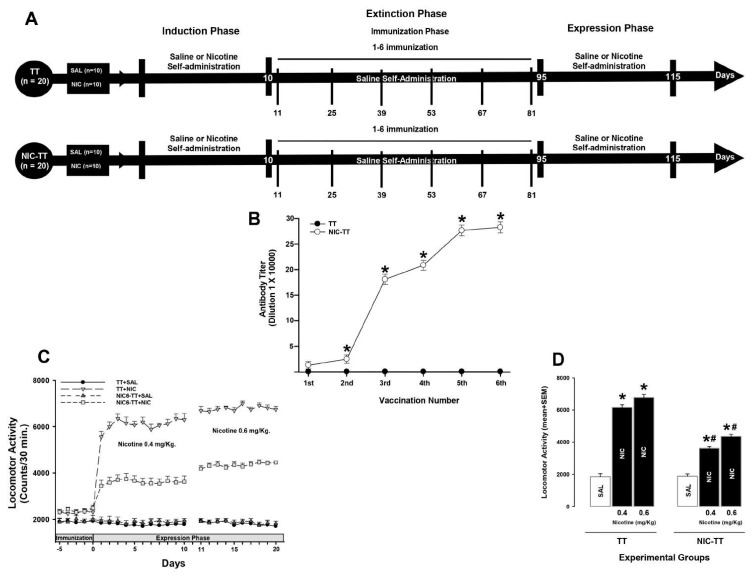
Experiment timeline (**A**). Antibody titer responses (to the sixth boost) in rats immunized with the TT or NIC_6_-TT vaccine (**B**). Mean titers (±S.E.M.). * *p* < 0.01 indicates significant effects of the antibody titers generated by the NIC_6_-TT vaccine following the sixth booster compared to the antibody titers generated by the TT vaccine in Wistar rats. The NIC_6_-TT vaccine attenuated the nicotine-induced locomotor activity (**C**). Mean locomotor activity (±S.E.M.) by group (n = 10 animals per group), measured during expression of locomotor sensitization (**D**). * *p* < 0.01 indicates significant effects on nicotine-induced locomotor activity in the TT + NIC and NIC_6_-TT + NIC groups compared to saline-treated groups. # *p* < 0.01 indicates significant effects in the nicotine-induced locomotor activity in the TT + NIC group compared to the NIC_6_-TT + NIC group, as determined by two-way ANOVA followed by Tukey’s tests.

This experiment was divided into three experimental phases. Phase I, the nicotine-induction phase, lasted 10 days. Phase II, the immunization phase, lasted 85 days. Phase III, the nicotine-expression phase, lasted 20 days (Figure 3A).

The saline groups received IP sterile saline solution (9% NaCl) during the three phases. During the immunization phase, rats in the TT groups were immunized with the TT vaccine, and animals in the NIC_6_-TT group received the NIC_6_-TT vaccine.

The animals of the nicotine groups received nicotine (0.4 mg/kg) during the induction. During the expression phase, the animals received different doses of nicotine (0.4 or 0.6 mg/kg). In the immunization phase, nicotine was withdrawn and the groups received saline. The rats in the TT groups received immunization with the TT vaccine and the animals in the NIC_6_-TT group received the vaccination with the NIC_6_-TT vaccine. Fourteen days after each immunization, serum samples were taken to assess antibody titers. All groups received 6 immunizations, one immunization every 14 days. After each administration, locomotor activity was recorded for 30 min for each animal (Figure 3A).

### 2.8. Statistical Analysis

Data are expressed as the means ± S.E.M. SPSS software version 21 (IBM, Armonk, NY, USA, 2021) was used to perform all statistical analyses. The level of statistical significance was set at *p* < 0.05.

In Experiment 1, results of antibody titers were analyzed using two-way repeated-measures ANOVA, where vaccination number was the repeated measures, and treatments (TT or NIC_6_-TT), as the between-subject factors; followed by a Tukey’s test for post hoc comparisons.

In Experiment 2, the number of active lever responses was the parameter used to estimate nicotine self-administration responses. A two-way ANOVA was used to compare the active and inactive lever responses between groups, with groups (saline, nicotine, and cocaine) and treatment (TT and NIC_6_-TT vaccine) as between-subject factors. If there was a significant F value for the interaction, a post hoc analysis of differences in responses between groups (*p* < 0.05) was performed using an additional Tukey’s test.

In Experiment 3, locomotor activity was measured by photobeam interruptions during the testing session. The results for locomotor activity in each group were analyzed with a two-way ANOVA with groups and treatment as the between-subject factors, followed by a post hoc analysis (Tukey’s test).

## 3. Results

### 3.1. Experiment 1

The TT group, immunized with the TT vaccine, did not show an increase in anti-nicotine antibody titers (*p* = 0.99). In contrast, the animals immunized with the NIC_6_-TT vaccine showed a progressive increase (F = (1,18) 956.230, *p* < 0.001) in anti-nicotine antibody titers measured by ELISA (Figure 1B).

Tukey’s test found significant differences in the antibody titers shown by the NIC_6_-TT groups compared to the TT (*p* < 0.001) group, from the second immunization. Additionally, Tukey’s test found that the maximum anti-nicotine antibody titer for the NIC_6_-TT groups was reached after the fifth booster (*p* < 0.001).

During the titer maintenance phase, animals immunized with the TT vaccine did not show an increase in anti-nicotine antibody titers, from 30 (*p* = 0.98) to 240 (*p* = 0.99) days after the last immunization. In contrast, animals that were vaccinated with the NIC6-TT vaccine showed a gradual decrease in anti-nicotine antibody titers (F = (1,18) 7925.024, *p* < 0.001). The post hoc test revealed significant differences in the levels of anti-nicotine antibodies at 30 (*p* < 0.001), 60 (*p* < 0.001), 90 (*p* < 0.001), 120 (*p* < 0.002), 150 (*p* < 0.002), 180 (*p* < 0.003), 210 (*p* < 0.003), and 240 (*p* < 0.003) days after the last immunization (Figure 1C).

### 3.2. Experiment 2

#### 3.2.1. Titers

As shown in Figure 2B, the TT group, immunized with the TT vaccine, did not show an increase in anti-nicotine antibody titers (*p* = 1). In contrast, the animals immunized with the NIC_6_-TT vaccine showed a progressive increase (F = (1,18) 751.069, *p* < 0.001) in anti-nicotine antibody titer measured by ELISA.

Tukey’s test found significant differences in the antibody titers shown by the NIC_6_-TT groups compared to the TT (*p* < 0.001) group, starting from the second immunization. Additionally, Tukey’s test found that the maximum anti-nicotine antibody titer for the NIC_6_-TT groups was reached after the fourth booster (*p* < 0.001).

#### 3.2.2. Nicotine Self-Administration

During the re-acquisition of nicotine self-administration, the TT + NIC group, which self-administered initially 0.04 mg/kg and subsequently 0.06 mg/kg of nicotine, shows a significant increase (two-way ANOVA; group X treatment interaction; F (1,80) = 359.750 *p* < 0.001) in the mean active lever press responses compared to the NIC_6_-TT + NIC group (0.04 mg/kg, *p* < 0.001; 0.06 mg/kg, *p* < 0.001). Additionally, the statistical analysis showed no differences between the TT + NIC and NIC_6_-TT + NIC (*p* = 0.98) groups when cocaine (1 mg/kg) was self-administered. This suggests that the anti-nicotine antibodies produced by the NIC-TT vaccine specifically recognize nicotine.

Concerning the groups that self-administered saline, the post hoc analysis found no difference between the TT + SAL and NIC_6_-TT + SAL (*p* = 0.97) groups (Figure 2C,D). Additionally, the post hoc test found differences in the responses on the active lever press showed by the TT + SAL and NIC_6_-TT + SAL groups compared to those shown by the NIC_6_-TT + NIC (*p* < 0.001) group, in both doses of nicotine evaluated.

In addition, two-way ANOVA found significant differences in the mean responses on the inactive lever in the groups x treatment interaction (F (1,80) = 15.447, *p* < 0.001). Tukey’s test found no differences between the TT + NIC and NIC_6_-TT + NIC groups when 0.04 mg/kg (*p* = 0.97), 0.06 mg/kg (*p* = 0.91) of nicotine, or 1 mg/kg (*p* = 0.87) of cocaine were self-administered; similarly, it did not find differences between the TT + SAL and NIC_6_-TT + SAL (*p* = 0.98) groups (Figure 2C,D).

### 3.3. Experiment 3

#### 3.3.1. Titers

The TT group immunized with the TT vaccine did not show an increase in anti-nicotine antibody titers (*p* = 0.98). In contrast, the animals immunized with the NIC_6_-TT vaccine showed a progressive increase (F = (1,18) 257.381, *p* < 0.001) in anti-nicotine antibody titer measured by ELISA (Figure 3B).

Tukey’s test found significant differences in the antibody titers shown by the NIC_6_-TT groups compared to the TT group in the second (*p* < 0.03), third (*p* < 0.001), fourth (*p* < 0.001), fifth (*p* < 0.001), and sixth (*p* < 0.001) immunization. Additionally, the post hoc test found that the maximum anti-nicotine antibody titer for the NIC_6_-TT groups was reached after the fifth booster (*p* < 0.001).

#### 3.3.2. Nicotine Locomotor Activity

As shown in Figure 3C, a dose of 0.4 mg/kg and 0.6 mg/kg of nicotine significantly increased locomotor activity (two-way ANOVA; group X treatment interaction; F (1,60) = 19.726, *p* < 0.001) during the expression phase in the rats immunized with the TT vaccine compared to the TT + SAL (*p* < 0.001) and NIC_6_-TT + SAL (*p* < 0.001) groups. Additionally, statistical analysis revealed significant differences in the nicotine-induced locomotor activity shown by the TT + NIC group dosed with 0.4 mg/kg of nicotine compared to when this group was administered 0.6 mg/kg of nicotine (*p* < 0.002). In contrast, the post hoc test found no differences in locomotor activity between the TT + SAL and NIC_6_-TT + SAL groups (*p* = 0.96).

Regarding the group immunized with the NIC_6_-TT vaccine, Tukey’s test found significant differences in the locomotor activity induced by 0.4 mg/kg and 0.6 mg/kg of nicotine compared to the locomotor activity shown by the TT + NIC group dosed with 0.4 mg/kg (*p* < 0.001) and 0.6 mg/kg (*p* < 0.001) of nicotine. Finally, Tukey’s test revealed differences in the nicotine-induced locomotor activity shown by the NIC_6_-TT + NIC group when dosed with 0.4 mg/kg of nicotine compared to the locomotor activity induced by 0.6 mg/kg (*p* < 0.002) of nicotine (Figure 3C,D).

## 4. Discussion

The effectiveness of nicotine vaccines has been supported by studies in rodents and primates [7,8,9,10,11,12,13]. These studies indicate that immunization of animals with nicotine vaccines generated specific antibodies capable of capturing nicotine within the blood vessels and preventing its entry into the brain [4,5,6]. This alteration in the pharmacokinetics of nicotine decreased the psychomotor and reinforcing effects induced by nicotine [9,10,11,12,13]. In humans, studies reported similar results [14,15,16,17,18,19,20]. The Nic-Qb (NIC002), NicVax, Niccine, and TA-NIC vaccines generated nicotine-specific antibodies; however, nicotine-specific antibody levels were low and/or variable and unable to maintain a long-term abstinent state [21,22,23,24,25,26,27,28]. Thus, it is imperative to develop new vaccines, synthesized with structural elements approved for human use, or, where appropriate, to improve the nicotine vaccines previously described and evaluated, so that they produce a greater amount of anti-nicotine antibodies and that antibody levels are sustained for long periods, even in the absence of re-immunization; or optimize vaccines that have shown efficacy in producing long-term antibodies against other drugs such as heroin or cocaine.

In this sense, the use of various methodologies aimed at improving and/or optimizing some of the structural elements that make up a vaccine to induce a strong immune response against nicotine has been reported [29,30,31]. Methodological strategies range from the generation or optimization of new haptens to the use of new adjuvants capable of simultaneously stimulating various immunological pathways, including the use of various carrier proteins, different types of spacer arms, different administration routes, and new formulations [29,30,31,32,33].

In this study, we evaluated the efficacy of the NIC_6_-TT vaccine, which was developed from the M_6_-TT vaccine. The M_6_-TT vaccine, formulated with aluminum as an adjuvant, demonstrated in various studies that it can produce high levels of serum antibodies (≈1:300,000) capable of recognizing heroin, morphine, and their metabolites and attenuating the antinociceptive, reinforcing, and motor effects induced by heroin or morphine [34,35,36,37,38,39,40]. In fact, we previously evaluated the possibility of generating a vaccine from another vaccine, changing only the hapten and maintaining the rest of the structural elements of the vaccine. We recently reported the efficacy of the COC-TT vaccine, which was developed from the M_6_-TT vaccine [41]. The COC-TT vaccine was able to produce anti-cocaine antibody titers of ≈ 1:650,000, which could attenuate the cocaine self-administration and place preference, as well as cocaine-induced *Fos* protein expression [41].

These results are in line with what was found in this study, where rats immunized with the NIC_6_-TT vaccine absorbed with aluminum hydroxide gel showed anti-nicotine antibody titers of ≈ 1:170,000, which could attenuate the psychomotor and reinforcing effects of nicotine.

Given that the position of the hapten allows a lesser or greater exposure of the hapten, several studies have suggested that the hapten and the position of the hapten to which it is coupled to the carrier protein are determining factors to induce a robust immune response. In this sense, studies in rodents reported that vaccines, coupled to the 6′ position of the nicotine to the carrier protein, such as the CMUNic-KLH [4,5], GK56-KLH [46], Nic-KLH [47], HexonAM1-KLH [33], and Nic-6-HA-TCC [48] vaccines, produced anti-nicotine antibody titers of ≈1:10,000. In this study, nicotine was coupled at the 6′ position, via a glycine arm, to the tetanus toxoid. Immunization with the NIC_6_-TT vaccine generated nicotine-specific antibody titers of 1:170,000 to 1:250,000, consistent with the aforementioned studies.

On the other hand, anti-nicotine vaccines, in which the carrier protein was coupled to other positions of the nicotine showed consistent results. In rodents, the 3′-AmNic-rEPA [6], NIC-KLH [12], and IP18-KLH [8,9] vaccines showed an improvement in anti-nicotine antibody levels; these were from ≈ 1:28,000 to 1:250,000 [10]. In humans, the vaccines NicVAX, TA-NIC, Niccine, NicQb, and SEL-068, where nicotine was coupled to the carrier protein in different positions, generated antibody levels of ≈1:45,000 to 1:250,000 [17,19,21,25,27,49].

This suggests that regardless of the position at which nicotine was coupled to the carrier protein, the antibody titers produced by the NIC_6_-TT vaccine are similar to the rest of the previously reported vaccines. However, a difference between the NIC_6_-TT vaccine with the rest of the previously reported vaccines is that the antibody titers reported by the NicVAX, TA-NIC, Niccine, NicQb, and SEL-068 vaccines decreased rapidly after the last immunization, this is seen as a decrease in the number of long-term tobacco abstinent patients [20,21,22,23,24,25,26,27,28]. In animals, to our knowledge, there are no reports related to the long-term maintenance of the titer of nicotine-specific antibodies. In this study, we report that animals immunized with the NIC_6_-TT vaccine showed antibody titers > 1:30,000 up to 240 days after the last immunization. Therefore, the immunogenic property of the NIC_6_-TT vaccine of generating high and sustained nicotine-specific antibody titers would lie in other structural elements of the NIC_6_-TT vaccine.

In general, the different anti-nicotine vaccines reported to date were synthesized by coupling nicotine to carrier proteins such as keyhole limpet hemocyanin (KLH), bovine serum albumin (BSA), recombinant cholera toxin B (rCTB), virus-like particles formed by the coat protein of the bacteriophage (Qb), and recombinant Pseudomonas aeruginosa exoprotein A (rEPA) formulated mainly with incomplete Freund’s adjuvant (FCA), CpG ODN (CpG), or aluminum hydroxide, among others, as adjuvants [4,5,6,17,18,19,20,30].

In this sense, the structural-molecular elements that make up the NIC_6_-TT vaccine, as well as its formulation, which bear a certain similarity to the structural elements and formulation of the CMUNic-KLH [4,5], GK56-KLH [46], Nic-KLH [47], HexonAM1 [33], Nic-6-HA-TCC [48], 3′-AmNic-rEPA [6], NIC-KLH [12], and IP18-KLH [8,9] vaccines, are mainly the hapten and the adjuvant used—nicotine in the 3′ or 6′ position and aluminum hydroxide—however, the NIC_6_-TT vaccine was synthesized by coupling 6′-nicotine, via a glycine arm, to tetanus toxoid. Therefore, the difference in antibody titers between CMUNic-KLH, GK56-KLH, Nic-KLH, HexonAM1-KLH, Nic-6-HA-TCC, 3′-AmNic-rEPA, NIC-KLH, and IP18-KLH and NIC_6_-TT conjugates may, in principle, be the carrier protein used—tetanus toxoid.

Tetanus toxoid (TT) is a highly effective and immunogenic protein. It is safe, well tolerated, and non-toxic; it contains many epitopes and one of its uses is as a carrier to deliver molecules to the CNS [50]. TT has been used in the development and evaluation of various immunoconjugates against illicit drugs such as heroin/morphine [36], cocaine [41], methamphetamines [51], and recently fentanyl [52]. In fact, various studies suggested that nicotine vaccines synthesized with TT as a carrier protein were superior to vaccines conjugated with other carrier proteins [27,32,53]. This suggests that TT could be one of the structural elements of the NIC_6_-TT vaccine that participates not only in the high production of antibodies against nicotine but also in the production of long-term nicotine-specific antibodies (sustainment).

Clinical studies determined that smoking abstinence was dependent on anti-nicotine antibody levels [18,19]. In a phase II clinical trial, it was shown that immunization with the NicVAX vaccine, which uses recombinant *Pseudomonas aeruginosa* exoprotein A, as a carrier [14], generated abstinence for 30 days, exclusively in patients who showed the highest anti-nicotine antibody levels [18]. Subsequently, a phase III clinical report, with the NicVAX vaccine, found no effect of the vaccine on the smoking abstinence rate at the end of the study [14,21].

In addition, phase II clinical studies with the NicQb or the TA-NC vaccine, which use a virus-like particle Qb as a carrier (Cytos Biotechnology) or the cholera toxin-B subunit as a carrier protein (Celtic Pharma), respectively, reported similar results [20]. Immunization with both vaccines did not improve smoking abstinence rates [20]. These studies mentioned that the low smoking abstinence rate was related not only to an insufficient antibody titer but also to the failure to sustain long-term nicotine-specific antibody levels and suggested that a durable antibody response for several months could protect people who have recently quit smoking and could prevent a smoker from trying a cigarette and could prevent smoking relapses.

On the other hand, smokers immunized with the Niccine vaccine, which uses TT as a carrier protein, reported smoking abstinence for 6–9 months after the last immunization [27]. These results are in line with those obtained in this study. The NIC_6_-TT vaccine shows maintenance of the anti-nicotine antibody titer for up to 240 days after the last immunization. This immunogenic property could help maintain smoking abstinence for long periods. These results support the participation of TT in the production and maintenance of levels of specific antibodies to nicotine.

The hypothesis of the importance of the TT as one of the structural elements of a conjugate vaccine that increases the immunogenic properties of a conjugate vaccine is supported by evidence. This includes studies on a new generation of carriers, known as nano vaccines, where nano scaffolds or nanoparticle platforms (tSVP, SWNHs; Lipid-polymeric hybrid NPs, lipid-PLGA hybrid) were designed that could increase the surface-volume ratio to increase antigen presentation, optimize hapten coupling, improve hapten spatial organization, and simultaneously show adjuvant activity and increase levels of anti-nicotine antibodies [30,53]. However, to our knowledge, these studies did not show long-term results. Even if they managed to sustain anti-nicotine antibody titers for several months, an important limitation of the use of these nanoparticle platforms is the long regulatory path that they must comply with to be used in any clinical trial. However, these results together are in line with what was found in our study, where we found that the NIC_6_-TT conjugate produces high titers of anti-nicotine antibodies and supports the important role of the carrier protein (TT) in the generation of high and sustained anti-nicotine antibody levels.

Additionally, in this study, we found an attenuation in the nicotine-induced reinforcing and psychomotor effects, seen as a decrease in nicotine self-administration and expression of nicotine locomotor sensitization. These results are in line with previous reports [6,9,10,11,12,13,33].

Previous studies reported that rodents immunized with the IP-18-KLH [9], 3′-AmNic-rEPA [10], and AM1-KLH [11] vaccines showed a decrease in nicotine self-administration. Certainly, in these types of studies, the relationship between antibody titers and the self-administered nicotine dose is an important factor. In these studies, anti-nicotine antibody titers ranged from 1:10,000 to 1:240,000, depending on the vaccine, which were able to attenuate the self-administration of 0.01 to 0.03 mg/kg of nicotine [9,10,11]. These results are in line with our results, where the NIC_6_-TT vaccine decreased the self-administration of 0.04 and 0.06 mg/kg of nicotine.

As mentioned, the 6′ position of nicotine has previously been used because it generates nicotine-specific antibodies [4,5,46,47,48]. In this sense, the anti-nicotine antibodies produced by the NIC_6_-TT vaccine were not able to recognize the cocaine molecule, given that when nicotine was changed to cocaine, the animals increased the seek for cocaine.

The dose of nicotine has an important value, as the dose of nicotine is related to the number of cigarettes that a human consumes in a unit of time. Various studies have reported that a dose of 0.03 mg/kg is equivalent to the dose of nicotine absorbed by two cigarettes in a human; on the other hand, a dose of 0.06 mg/kg of nicotine is equivalent to smoking three cigarettes [5,6,10,31]. In this sense, the anti-nicotine antibodies produced by the NIC_6_-TT vaccine can reduce the reinforcing effect induced by three cigarettes.

However, a smoker can consume a greater number of cigarettes per day and, therefore, increase the concentration of nicotine in blood vessels. Therefore, it is necessary to evaluate the capacity of the anti-nicotine antibodies induced by the NIC_6_-TT vaccine in pharmacological models that require a range of higher doses of nicotine, such as nicotine-induced locomotor activity.

In this study, we found that the anti-nicotine antibodies produced by the NIC_6_-TT conjugate could reduce the expression of nicotine locomotor sensitization induced by 0.4 and 0.6 mg/kg of nicotine. These results are in line with a study that showed that male rats immunized with the NIC-KLH [11], 3′-AmNic-rEPA [6,12], and HexonAM1-KLH [32] vaccines exhibited a significant decrease in locomotor activity induced by 0.3 and 0.5 mg/kg of nicotine. These results suggest that the anti-nicotine antibodies produced by the NIC_6_-TT vaccine were sufficient to diminish the effect of high doses of nicotine.

### Future Perspectives

As mentioned above, smoking is a disease that causes millions of deaths each year and for which no effective therapy has been found. Active vaccination could certainly be a useful therapeutic tool, which would assist treatments that have shown some efficacy to date. However, for active vaccination to be successful, it must meet certain properties. (1) The nicotine vaccine must be synthesized with structural elements for human use so that it can be transferred to clinical evaluations quickly; (2) it must generate high and sustained levels of specific antibodies; and (3) the antibodies must be able to attenuate the effect of various doses of nicotine, decreasing the reinforcing effects of nicotine. In this sense, the literature indicates that vaccines synthesized using tetanus toxoid as a carrier protein and formulated with alumina as an adjuvant have been shown to meet these three properties. The NIC_6_-TT vaccine is no exception. Therefore, rather than seeking new nano-molecular elements and the evaluation of new adjuvants, research should be aimed at improving vaccines that have already shown efficacy and that are synthesized with elements for human use, such as NIC_6_-TT, so that in the short term, there is an effective model that can reduce the harmful effects of smoking.

## 5. Conclusions

An important limitation regarding a proposal to transfer the NIC_6_-TT vaccine to clinical protocols is related to the number of immunizations required to reach the maximum levels of antibodies (six immunizations), given that clinical trials have indicated that nicotine-dependent patients show poor adherence to treatment [16,18,20,23,24,26,48]. Despite this limitation, the results of this study suggest that at the dose of vaccine and the dose of nicotine used in the pharmacological evaluations, the antibody titers generated by the NIC_6_-TT vaccine are capable of attenuating the reinforcing and psychomotor effects of nicotine.

On the other hand, currently, the main issue in the field of nicotine vaccines is not to generate vaccines that produce high titers of antibodies capable of reducing the reinforcing and psychomotor effects induced by different doses of nicotine. The most relevant issue is that the levels of antibodies produced by vaccines do not decay rapidly and are sustained in the long term, to maintain smoking abstinence. As far as this study is concerned, the NIC_6_-TT vaccine met three important properties: high titers of nicotine-specific antibodies, sustained antibody levels for up to 240 days, and antibodies that decrease the reinforcing and psychomotor effect of different doses of nicotine. Collectively, these results support its potential use in clinical studies for the treatment of smokers.

## Data Availability

The data supporting the findings of this study are available upon reasonable request from the corresponding author, due to the research policies of the National Institute of Psychiatry. Products intended for certain uses in humans must be protected, and their availability for other uses depends on the authorization of the authorities of the National Institute of Psychiatry to the researcher.

## Supplementary Materials

The following supporting information can be downloaded at: https://www.mdpi.com/article/10.3390/brainsci15040364/s1. Refs. [54,55,56,57] are cited in Supplementary Materials.
