# Supplementary material for: NIC6-TT Vaccine Reduces Nicotine-Seeking Behavior and Expression of Nicotine-Induced Locomotor Sensitization in Rats
Source: Brain Sci. 2025 Mar 31;15(4):364. doi: 10.3390/brainsci15040364 (PMC12026458; doi:10.3390/brainsci15040364)
Supplement: Supplementary file 1 [file brainsci-15-00364-s001.zip › brainsci-3379934-supplementary.pdf]

Supplementary Material.

2.3 Synthesis of the NIC-TT vaccine

The 6' position of nicotine was selected to bind to the spacer arm because previous studies demonstrated that binding of the spacer arm to the 6' position generated nicotine-specific antibodies [4,5]. The synthesis of the NIC-TT vaccine began with the production of the intermediate 6'-nicotine-glycine (6'-NIC-GLY) as described by Pravetoni et al., 2012 [42] Once the 6'-NIC-GLY intermediate was synthesized, it was activated for coupling with water-soluble 1-(3-dimethyl aminopropyl)-3-ethyl carbodiimide (EDC, Pierce, Rockford, IL, USA).

To prepare the TT-TFCS conjugate (tetanus toxoid+N-(ε-trifluoroacetyl caproyloxy) succinimide ester), the TFCS (Pierce, Rockford, IL, USA) was dissolved in a freshly prepared solution of 10–20% DMSO (Sigma–Aldrich, St. Louis, MO, USA)/80% distilled H2O. This solution was mixed with the tetanus toxoid (TT) at a volume of 4ml of PBS, pH 7.2, and incubated at room temperature overnight. To remove the TFCS trifluoroacetyl protecting group, which is required to couple the TFCS to the ε-amino groups of the side chain of lysine residues of the TT, it was incubated at pH 8.1 in PBS at room temperature for up to 3 hours. The final purification of the TT-TFCS derivative was carried out by exhaustive dialysis against PBS, pH 7.2.

To prepare the NIC_6-TT conjugate, the activated EDC-6'-NIC-GLY was added to TT-TFCS in a volume of 100 ml of PBS, pH 7.5, and the reaction mixture was incubated under gentle stirring at room temperature overnight. After exhaustive

dialysis against PBS, pH 7.4, the purified conjugate was concentrated by pressure dialysis, aliquoted (unit dose=1mg TT/ml), and stored in sealed sterile glass vials. The NIC BSA conjugate was synthesized using the same method as for TT.

2.4 Determination of serum antibody titters via ELISA

A solid-phase antibody capture ELISA was used to monitor antibody titer responses after each booster in vaccinated animals. ELISA plates were coated with NIC_6-BSA conjugate, and a range of serial dilutions of antisera was added in triplicate. Biotin-labelled anti-mouse secondary antibody (Jackson Immunoresearch, West Grove, PA, USA), coupled to an OPD system (Sigma–Aldrich, St. Louis, MO, USA) as chromogenic substrate, was used to detect immunopositive signals. Antibody titters were initially defined as the inverse of the serum dilution that gave 50% of the maximal response.

2.5.1 Self-Administration Procedure

2.5.1.1 Apparatus

For each experiment, we used an operant Skinner box (30 × 28 × 30 cm; TSE Germany) equipped with a house light, a ventilation fan, a drug-infusion pump, a fluid swivel attached to a counterbalance arm, a light-cue panel above each lever, and a food-pellet dispenser between the two levers (**Figure A**). The levers were 9 cm above the grid floor, but only one of them, the retractable (active) lever, was

operational during drug delivery. Each experimental chamber was placed into a wooden box with sound-attenuating insulation **[41,43,54].**

2.5.1.2 Surgery

For surgery, the rats were anesthetized with ketamine HCl (90 mg/kg, i.p. Sigma Aldrich) and xylazine (5 mg/kg, i.p. Sigma Aldrich). A surgical incision (5 mm) was made above the jugular vein and the vein was located by dissection. For catheter implantation, a connector pedestal (20 ga 300-001; Plastics One, Wallingford, CT, USA) attached to a propylene catheter (0.51 mm ID, 0.94 mm OD; SILASTIC) was glued to a ProLite mesh (2-cm diameter; Silicon Polypropylene Mesh, Wall, USA) with dental cement. The end of the tubing attached to the connector pedestal was inserted subcutaneously into the area of the right jugular vein and then 3.0 cm into the vein. It was secured with silk sutures. The other end of the catheter tubing was implanted subcutaneously between the shoulder blades of the animals.

Wounds were treated with nitrofurazone and antibiotic ointment. Patency of the catheter was maintained by flushing it daily (Sunday through Saturday), after self-infusion sessions, with a mixed solution consisting of 0.1 ml saline solution (0.9% NaCl, Sigma Aldrich)/12.5 IU heparin (Pisa Agropecuaria Mexico)/100 mg/ml gentamicin (Schein Pharmaceuticals, USA). Prior the self-administration sessions, implanted animals received 0.1 ml heparinized saline (10 IU/ml). During the self-administration procedures, the catheters attached to the pedestal guide cannulas were connected to an infusion-pump system (PHM-100, Med-Associates) through a three-channel fluid swivel (TSE System, USA) fixed above the operant-conditioning chamber.

2.5.1.3 Procedures

2.5.1.3.1 Lever-Press Training

Before surgery, the rats were trained to lever press for 45-mg food pellets (Noyes, Lancaster, NH, USA) under a fixed-ratio 1 (FR1) schedule of daily 2-h sessions/6 days per week, with light stimulus presentation that had indicated food delivery during training (Fig. A). Food delivery was controlled by computer software (TSE Systems, Hamburg, Germany). The rats that learned to lever press for 35 food pellets received further catheter-implantation.

2.5.1.3.2 Nicotine Self-Administration Training

The rats were trained to self-administer nicotine using a unit dose-infusion schedule of 0.04 mg/kg nicotine (30 µl/infusion), in daily 2-h sessions during 6 days per week. The maximum number of infusions per session was always 35 infusions. During the initial 10 days, rats acquired nicotine self-administration under an FR1 schedule of reinforcement; then rats acquired nicotine self-administration under an FR3 schedule for 3 days followed by an FR5 schedule for 5 days. Once the FR5 ratio was reached and the animal achieved 30-35 infusions per session, a 120-sec TO, was implemented following the delivery of each reinforcer. The house light remained on throughout each daily session. Completion of the reinforcement schedule on the active lever produced the simultaneous activation of a cue light above the active lever followed by an i.v. infusion of nicotine (0.04 mg/kg/infusion); immediately after each infusion, other cue light above the lever was activated

signalling a 120-s timeout period. This light remained illuminated for a 120-s time-out (TO) period during which responses were recorded but did not have any programmed consequences. All the rats were exposed to this FR5 120-sec TO schedule for 10 sessions until meeting the criterion for the acquisition of nicotine self-administration. The criterion was defined as stable nicotine-maintained responding with 30-35 infusions per session, for 10 consecutive days, and less than 10% variability in the number of infusions, for at least 5 consecutive days. Only the animals that met this criterion were used for subsequent experimental procedures (Fig A).

2.5.1.3.4 Extinction

Extinction sessions began after achieving baseline response rates for nicotine delivery**.** The experimental conditions were the same as during nicotine self-administration with the exception that that pressing on the active lever resulted in the infusion of saline instead of nicotine; the houselights signalled the initiation of sessions and remained "on" throughout each daily 2-h session/6 days per week, and responses on the lever previously associated with nicotine infusions activated the sound of the infusion pump and the stimulus lights directly above the lever. Responses on the inactive lever again had no consequences. Rats exhibiting extinction response rates of ≤30% compared to the nicotine baseline responses obtained during the training period were removed from the experiment (Fig A).

2.5.1.3.5 Re-acquisition

The nicotine or cocaine self-administration procedures during the re-acquisition sessions were similar to those used during training for nicotine self-administration (FR5 with 2-min TO). Responses on the active lever were recorded for each animal.

2.5.2 Behavioural Sensitization Procedure

For each animal, locomotor activity was assessed in transparent Plexiglass activity chambers (50x50x30 cm) linked to a PC. Each activity chamber was surrounded by a 16x16 photocell beam array located 3 cm from the floor surface to scan locomotor activity (OMNIALVA, Instruments, Mexico; **Figure C**). Photobeam interruptions were automatically quantified with OABiomed software (1.1) and analysed afterward **[44,55-57]**. Locomotor activity was defined as the interruption of consecutive photobeams (OMNIALVA, Mexico).

2.5.3 Procedure

The animals were habituated to the activity chambers in three 30-minute sessions and were randomly assigned to different pharmacological treatment groups. Locomotor activity was recorded for 30 minutes. The rats were returned to their home cages after each experimental session had been completed.

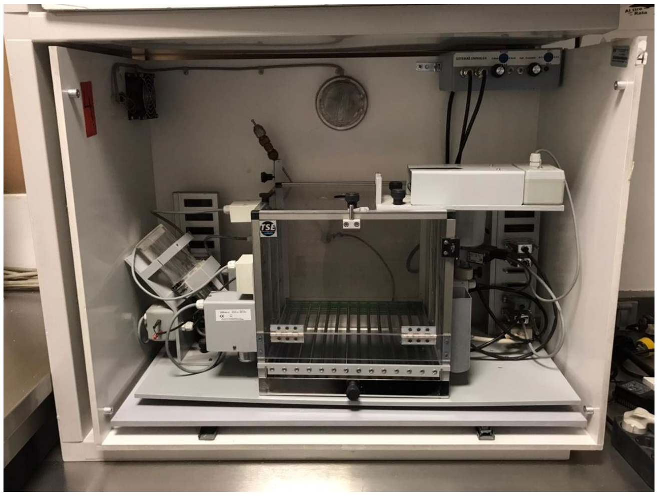

FIGURE A

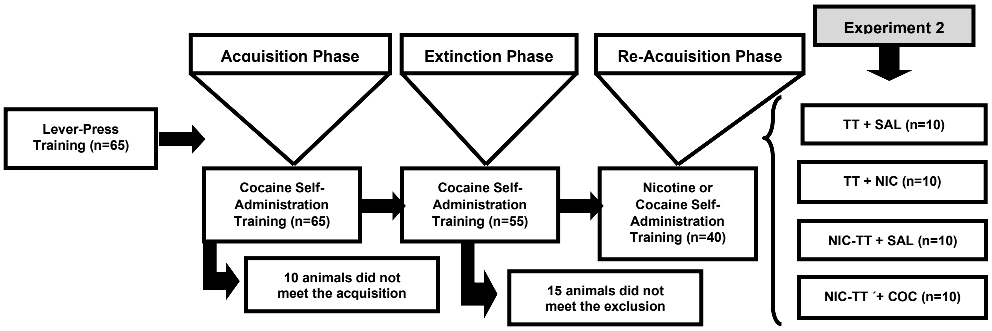

FIGURE B

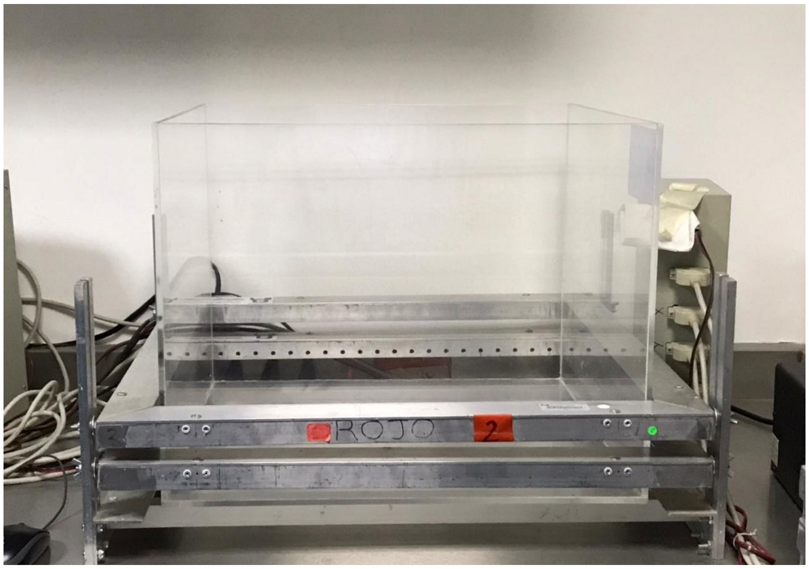

FIGURE C
